# Teachers' perception of aggressive student behavior through the lens of chronic worry and resignation, and its association with psychophysiological stress: An observational study

**DOI:** 10.1007/s11218-023-09782-2

**Published:** 2023-04-06

**Authors:** Alexander Wettstein, Gabriel Jenni, Sandra Schneider, Fabienne Kühne, Martin grosse Holtforth, Roberto La Marca

**Affiliations:** 1 Department of Research and Development, University of Teacher Education Bern, Bern, Switzerland; 2 Clinical Psychology and Psychotherapy, Department of Psychology, University of Bern, Bern, Switzerland; 3 Psychosomatic Medicine, Department of Neurology, Inselspital, Bern University Hospital, University of Bern, Bern, Switzerland; 4 Centre for Stress-Related Disorders, Clinica Holistica Engiadina, Susch, Switzerland; 5 Clinical Psychology and Psychotherapy, Department of Psychology, University of Zurich, Zurich, Switzerland

**Keywords:** Student aggression, Teacher stress, Coping styles, Vital exhaustion, Hair cortisol, Behavioral observation

## Abstract

Aggressive student behavior is considered a leading risk factor for teacher stress. However, teachers' coping styles may affect how they perceive and respond to aggressive student behavior. This study tests whether teachers' perceptions of aggressive student behavior mainly mirror objectively observed aggression in presence of the teacher (as coded by external observers) or whether teachers' perception of aggressive student behavior primarily reflects teachers' avoidant coping styles, such as chronic worry and resignation. Finally, we examine whether observed and teacher-perceived aggression relates to increased vital exhaustion and psychophysiological stress among teachers (i.e., higher hair cortisol concentration). In an ambulatory assessment study, we administered self-reports to 42 Swiss teachers to assess perceived student aggression, chronic worry, resignation, and vital exhaustion. Additionally, four consecutive lessons per teacher were filmed, and aggressive student behavior in presence of the teacher was coded by four trained external observers. The concentration of cortisol was assessed in hair samples. Results showed that teacher-perceived and observed aggression were moderately associated. Observed aggression was related to teacher perceptions to a much lesser extent than teachers' avoidant coping styles, that is, chronic worry and resignation. While teacher-perceived student aggression was associated with teachers' self-reported vital exhaustion, we did not find any significant association with hair-cortisol concentration. Our findings suggest that teachers perceive student aggression through the lens of their coping styles. Teachers' dysfunctional coping styles are associated with an overestimation of student aggression. Teachers' overestimation of student aggression relates to higher levels of vital exhaustion. Therefore, it is crucial to identify and change teachers' dysfunctional coping styles to prevent a vicious cycle of dysfunctional teacher–student interactions.

## Introduction

### Aggressive student behavior as a crucial source of teacher stress

Comparative studies from Germany, the United Kingdom, and the Netherlands show that teachers report higher levels of self-perceived workplace stress (Smith et al., 2000; Unterbrink et al., 2007) and higher rates of burnout (Schaufeli, 2003) compared to other professions. One primary source of teachers' stress is student misbehavior (Dicke et al., 2014; McCormick & Barnett, 2011), which increases the risk of burnout among teachers (Kokkinos, 2007; Tsouloupas et al., 2010) and also represents a significant reason for teacher attrition (Ingersoll, 2003). For teachers, student misbehavior seems particularly stressful when it turns into aggressive behavior (Lehr, 2004). Aggressive behavior refers to any behavior performed with the intent of causing physical or mental harm to another person who is motivated to avoid the harm (e.g., Anderson & Bushman, 2002; Wettstein, 2008). Aggressive behavior at school takes many forms: verbal or physical, direct (e.g., insulting, hitting) or indirect (e.g., hiding objects, spreading rumors; Björkqvist et al., 1992), and has far-reaching negative consequences, namely for students' social and cognitive development, teaching quality and the teacher's health. Chang and Davis (2009) point out that aggressive student behavior may be the most challenging problem teachers face in the classroom. Thus, it can be assumed that aggressive student behavior is one of the primary sources of teacher stress in the classroom, with possible adverse long-term effects on teachers' health.

However, most studies have assessed aggressive student behavior using teacher ratings rather than systematic behavioral observation by trained external observers. Studies on student misbehavior show a moderate agreement between teachers' and observers' perspectives (*r* = .35 – .42) (Achenbach et al., 1987; Skiba, 1989). A more recent study on student aggression (Scherzinger & Wettstein, 2019) found only a weak average agreement of *r* = .24 between observed and teacher-perceived student aggression. The discrepancy between teacher and observer perspectives may have various reasons. First, teachers are in a difficult observer position. In the classroom, teachers face complex social situations and have to fulfill multiple tasks at the same time. This makes it difficult for them to perceive social processes in the classroom as accurately as external observers (Wettstein, 2008). Second, teachers' personality traits, such as emotional lability, may be associated with an increased perception of aggressive student behavior (Evers et al., 2004; Kokkinos et al., 2005; Tsouloupas et al., 2010). Third, prior stress experience may also affect teachers' perceptions. Accordingly, previous studies have found links between teachers' emotional exhaustion and an increased perception of student misbehavior (Evers et al., 2004; Kokkinos et al., 2005; Tsouloupas et al., 2010). Thus, teacher stress is not exclusively an objective reflection of external stressors but also reflects teachers' personalities, prior stress experiences, and their coping styles (Chan, 1998).

Although there is a broad consensus that aggressive student behavior is a crucial source of teacher stress, many questions remain. Prior research has relied primarily on teacher reports of student aggression and teachers' stress experience. It remains unclear whether (a) it is primarily teacher-perceived or objectively observable aggressive behavior that relates to teacher stress and (b) whether teacher-perceived student aggression is biased by their own dysfunctional coping styles, resulting in an overestimation of aggressive student behavior. The present study addresses these open questions, explores teachers' perceptions of aggressive student behavior through the lens of chronic worry and resignation, and examines the association between perceived aggression and psychophysiological stress in the teachers' natural settings. This study's findings may help reduce teacher stress by sensitizing teachers to potential avoidant coping styles biasing their perception, and fostering the proactive use of problem-focused coping styles.

### Avoidant coping styles

Coping appears to play a crucial role in how individuals perceive potentially stressful situations (Serwinski, 2017). Coping styles range from active/problem-focused to avoidant/emotion-focused (Finset et al., 2002). Active/problem-focused coping styles may mitigate teacher stress, whereas avoidant/emotion-focused coping styles may increase teachers' stress experience, especially when assuming that most stressors are manageable.

Chronic worry is an avoidant, emotion-focused coping style and refers to persistent anxious apprehension of possible future adverse events and is associated with negative emotions (Barlow, 2002). Affected individuals describe chronic worry as an uncontrollable chain of negative thoughts and images. Lewis (1999) found that teachers who worry more about classroom discipline and student misbehavior report above-average dysfunctional coping styles. Chronic worry results from a lack of adaptive emotion-regulation strategies to temporarily avoid physiological arousal and negative emotions (Borkovec et al., 2004; Mennin et al., 2002). Individuals who worry more show higher levels of physiological arousal before and after a stress-inducing situation (Newman & Llera, 2011). Additionally, individuals who worry more experience stress episodes for longer and more intensely than those who worry less (Schulz et al., 2004). Chronic worry also increases social stress and sleep problems (Schulz et al., 2003). In sum, chronic worry has been found to result in high psychological costs and is strongly associated with self-reported stress (Szabó, 2011). In addition, chronic worry has also been associated with the salivary biomarker cortisol, specifically, with an increased cortisol awakening response (Wüst et al., 2000). Hair cortisol concentration (HCC) reflects long-term cumulative hair cortisol levels and thus presumably indicates chronic stress exposure (Russell et al., 2012). Consequently, it can be assumed that an increased HCC may also reflect chronic worry.

Passive resignation is also an avoidant, emotion-focused coping style used when individuals believe they have no control and are unable to make a difference (Finset et al., 2002). Consequently, people with an emotion-focused coping style quickly resign and give up (Schaarschmidt & Fischer, 2008), potentially reducing their stress resilience. Contrary to other coping styles, resignation has been associated with a lower sense of personal accomplishment and professional success (Schorn & Buchwald, 2006). In a teacher intervention study, Braeunig et al. (2018) found that a decrease in resignation improves teachers' general mental health.

In sum, avoidant, emotion-focused coping styles may exacerbate teacher stress, which in turn may lead to the experience of more psychological symptoms (Chan, 1998). For example, teachers with avoidant, emotion-focused coping styles show higher burnout levels than teachers with active, problem-focused coping styles (van Dick & Wagner, 2001). Finally, dysfunctional coping styles are associated not only with an increased stress experience but also with teachers' classroom behavior. Tran (2016) found that teachers who use passive avoidant coping styles employ more aggressive and punitive techniques in response to student misbehavior. Avoidant coping styles such as chronic worry and passive resignation may reduce stress resilience in the long run, as the underlying problems causing stress remain unresolved. Thus, avoidant coping may deplete teachers' resources and contribute to their long-term stress experience.

On a psychological level, we distinguish two stress-related outcomes: emotional exhaustion and vital exhaustion. Emotional exhaustion is defined as feelings of emotional overstrain and reduced emotional resources (Maslach & Jackson, 1986). Vital exhaustion refers to a state of excessive fatigue, lack of energy, increased irritability, sleep disturbances, loss of libido, and feelings of demoralization and is considered a potential early warning sign of cardiovascular disease (Appels & Mulder, 1989). Avoidant coping styles have been associated with emotional exhaustion (Ito & Brotheridge, 2003), vital exhaustion (van Zijderveld et al., 2013), and elevated HCC (Serwinski, 2017). In contrast, active, problem-focused coping styles are negatively associated with emotional exhaustion (De Rijk et al., 1998).

### Psychophysiological stress consequences

When we perceive a situation as potentially threatening, we show psychological, physiological, and behavioral stress responses (Lazarus, 1991; Mauss & Robinson, 2009). So far, research on teacher stress has focused on their psychological stress responses, neglecting physiological stress reactions. Physiologically, acute stress prepares the body for a challenge and is not harmful in the short term (Birbaumer & Schmidt, 2010). Chronic stress, however, endangers teachers' health and well-being by potentially leading to long-term physiological (e.g., altered autonomous nervous system activity, hypothalamic–pituitary–adrenal axis dysfunctions, subclinical inflammation), psychosomatic (e.g., sleep disorders, depressiveness, anxiousness, Bellingrath et al., 2008; Qi et al., 2014), and social (e.g., withdrawal, social insecurity) stress consequences (Klusmann et al., 2016; Kokkinos, 2007).

On a psychosomatic level, chronic stress consequences can be assessed by self-rated vital exhaustion. Whereas the process of vital exhaustion is not yet fully understood, it may result from a failed adaptation to chronic stress (Schoch et al., 2018) and is closely related to the concept of burnout. Women generally report higher levels of vital exhaustion than men, which may be due to psychological and physiological sex differences in the stress response (Frestad & Prescott, 2017; Prescott et al., 2003; Verma et al., 2011). Teachers appear to be at an increased risk of vital exhaustion compared to other professions (Guglielmi & Tatrow, 1998). Kudielka et al. (2008) found an increased risk of vital exhaustion in a sample of 150 male and female school teachers. Bellingrath et al. (2008) found in a sample of 104 female school teachers that 47% reported being highly exhausted.

On a physiological level, chronic stress exposure can be reflected by the HCC, a relatively new approach to measure long-term cumulative cortisol levels (Russell et al., 2012). Systemic cortisol exposure during specific periods can be analyzed by segmenting the corresponding hair sample. Previous studies on the relationship between self-reported stress and HCC have been inconsistent. In about half of the studies, no significant associations were found (Gidlow et al., 2016; Kudielka et al., 2006; Rohleder, 2018). Regarding work stress, a study of 39 Chinese kindergarten teachers (Qi et al., 2014) showed a significant positive association between experienced effort–reward imbalance at work and HCC.

### Present study

This study is part of a larger ambulatory assessment project on stressful interactions in the classroom (Wettstein et al., 2021). The present study aims to investigate (1) to what extent teachers' perceptions of aggressive student behavior correspond to the codings of trained external observers and (2) whether teachers perceive student aggression through the subjective lens of their own avoidant coping styles (i.e., chronic worry and resignation). Finally, we examine (3) how teacher-perceived student aggression is associated with chronic stress consequences (i.e., vital exhaustion and HCC).

Based on previous research on the agreement of teacher and observer agreement on aggressive student behavior (Achenbach et al., 1987; Skiba, 1989), we expect that observed student aggression in the presence of the teacher and teacher-perceived student aggression are only moderately associated (Hypothesis 1). Previous studies indicate that avoidant coping styles may exacerbate teacher stress. We, therefore, expect that chronic worry and resignation are related to teacher-perceived student aggression. We assume that these dysfunctional coping styles are associated with overestimating the occurrence of student aggression (Hypothesis 2).

Given that coping style plays a crucial role in the impact of stress on the individual, we expect that teacher-perceived and, to a lesser extent, observed student aggression is significantly associated with vital exhaustion (Hypothesis 3). We are unaware of any studies on HCC and teacher-perceived student aggression, making accurate predictions challenging. Furthermore, previous studies on the relationship between self-reported stress and HCC have been inconsistent. Nevertheless, we expect that teacher-perceived aggression is significantly associated with HCC (Hypothesis 4).

## Method

### Participants

Participants were recruited via flyers and circular emails. The inclusion criterion for participation in the study was an occupation as a primary or secondary teacher and a workload of a minimum of 16 lessons per week (equivalent to at least a 60 percent occupation). Exclusion criteria were working outside of the canton of Bern (Switzerland), acute infections, cardiovascular or other chronic diseases, use of cardiovascular drugs or other medication in the past two months (besides herbal medicine), substance abuse, consumption of psychoactive substances in the last four weeks, more than two alcoholic standard beverages per day, smoking more than ten cigarettes per day, having taken a long-distance flight within the last two weeks, and pregnancy. All teachers were screened in a short interview to ensure that they met inclusion and exclusion criteria. Initially, 76 teachers expressed their willingness to participate in the study. Twenty-one could not participate due to the following exclusion criteria: chronic diseases (*n* = 8), medication (*n* = 5), contact termination (*n* = 4), living and working outside the canton of Bern (*n* = 1), teaching less than 16 lessons per week (*n* = 1), pregnancy (*n* = 1), and emigration from Switzerland during data collection (*n* = 1). Before collection started, *n* = 7 withdrew their consent. Of the 48 teachers completing the online questionnaire, *n* = 6 decided to discontinue their participation, resulting in a final sample size of *n* = 42 teachers (28 females; mean age = 39.66, *SD* = 11.99, range = 23–63). On average, they had 13.35 years of teaching experience (*SD* = 11.07, range = 1–40). Twenty-seven teachers (64.29%) taught in kindergarten and elementary school (1st to 6th grade), twelve (28.57%) in secondary school (7th to 9th grade), and three (7.14%) in high school and vocational school (10th to12th grade). Three participants were either bald or had too short hair to take a hair sample. One participant was excluded due to using a hair product containing cortisone, resulting in 38 teachers referring to hair markers. Participants all signed informed consent. The study was conducted in strict compliance with current national data protection laws and approved by the Ethics Committee of the Canton of Bern.

### Design

This multimodal study combines self-reports, physiological measures, and systematic behavioral observation of aggressive student behavior in the classroom to study the participants in their "natural habitats" (Trull & Ebner-Priemer, 2013) and increase ecological validity.

### Procedure

Teachers provided information on demographic variables (sex, age, years of teaching experience, number of lessons taught per week) and completed an online questionnaire on perceived student aggression, chronic worry, resignation, and vital exhaustion. Subsequently, our participants came to the University of Bern, where hair samples were taken. Research assistants then visited the participants' schools to record four consecutive lessons of 45 minutes each.

### Measures

#### Self-reports

*Teachers' perception of student aggression *was assessed globally with the Classroom Questionnaire (Wettstein et al., 2016). Sample items are: *"Children hit or kick other children," "Children deliberately accuse other children, even though they have not done anything." *Items were rated on a five-point Likert scale from "never" (1) to "very often" (5). Mean values were calculated, and Cronbach's alpha of this four-item scale was α = .91.

*Chronic worry *was assessed with the respective subscale of the Trier Inventory for Chronic Stress (TICS; Schulz et al., 2004). The subscale consists of four items and measures the frequency of worry. Sample items are: "*There are times when I worry a lot, and I cannot stop*." "*I have a fear that something unpleasant will happen."* Each item was rated on a five-point Likert scale from "never" (1) to "very often" (5). Mean values were calculated, and Cronbach's alpha was α = .92.

*Resignation tendency *was assessed with the Resignation Tendency subscale of the Measure of Coping Capacity Questionnaire (MECCA; Schaarschmidt & Fischer, 2008). Participants rated the six items on a five-point Likert scale, with values between "absolutely disagree" (1) to "absolutely agree" (5). Sample items are *"If I fail, I resign quickly." "I easily lose heart when I do not succeed despite the effort." *This resulted in a total sum score ranging from 6 to 30 points. Cronbach's alpha was α = .84.

*Vital exhaustion* was assessed with the German translation (cf. Rau et al., 2010) of the Maastricht Vital Exhaustion Questionnaire (MQ; Appels et al., 1987). The scale consists of 21 items assessing fatigue, difficulties falling asleep, general malaise, apathy, irritability, energy loss, depression, and waking up exhausted. Sample items were: *"Do you sometimes feel as if your body is like a battery that is losing its power?", "Do you have increasing difficulty in concentrating on a single subject for long?"* The 21 items can be answered with "no", resulting in a score of 1 in our study; undetermined, which is marked with "?" and scored as 2; and "yes", scored as 3. By summing the scores of each item, the total score can be calculated, with higher scores indicating greater vital exhaustion. Cronbach's alpha was α = .88.

#### Observed aggression

Classrooms were equipped with GoPro cameras and microphones for video observation. Four lessons of 45 minutes each were recorded in each class after a three-day acclimatization period to reduce potential reactivity. Aggressive student behavior in presence of the teacher was coded and analyzed by four trained external observers in an event-sampling procedure using the BASYS observation system (Wettstein, 2008). The observers and the teachers assessed the same group of students. Prior to coding, all four observers (research assistants) were trained to a criterion of .80 (Cohen's kappa), and unclear episodes were discussed regularly by the observer team. Video coding was performed using MAXQDA Analytics Pro 2020 version 20.4.1 (VERBI Software, 2019). To determine inter-rater reliability, 11% of the coded video material was recoded by another observer, resulting in a Cohen's kappa coefficient of .85. Mean frequencies of aggressive student behavior for each lesson (45 min.) were calculated when the teacher was present in the classroom and likely to notice aggressive student behavior.

#### Hair cortisol concentration

The amount of cortisol in the hair provides information about how much a person is burdened by persistent stress. The longer the stress lasts, the longer an increased concentration of cortisol circulates in the body—and more cortisol accumulates in the hair. On average, hair grows one centimeter per month. Hair cortisol concentration during the last three months was measured on a three-centimeter-long hair strand. Hair strands were cut from the posterior vertex as close to the scalp as possible. HCC was determined from the three centimeter segment closest to the scalp. Given an average hair growth of 1 cm per month (Wennig, 2000), this segment represents the cumulative glucocorticoid secretion over three months before sampling. The washing procedure and glucocorticoid extraction followed the laboratory protocol described by Gao et al. (2013). All samples were analyzed by liquid chromatography coupled with tandem mass spectrometry (LC–MS/MS). This analysis's lower limits of quantification (LOQ) were below 0.1 pg/mg for cortisol. The inter- and intra-assay coefficients of variance for cortisol were below 15% (Gao et al., 2013).

#### Data analysis

For all scales, there were no missings or imputations. The Shapiro–Wilk test was used for each variable to test whether the data were normally distributed. The variables observed and perceived aggressive student behavior, chronic worry, vital exhaustion, HCC, age, and years of work experience were log-transformed due to non-normal distribution. Therefore, all correlations and regressions involving these variables were calculated and interpreted using this transformation. Multicollinearity diagnostics were conducted and revealed correlations within an acceptable range. Participants' sex, years of work experience, and the number of lessons taught per week were controlled for in the models, where appropriate (i.e., when significantly related to the dependent and/or independent variable). The assumptions of multiple linear regression were tested and not violated.

All analyses were conducted using IBM SPSS Statistics version 28. Descriptive statistics and bivariate correlations were computed to investigate the study's variables. The research questions were analyzed by simple and multiple linear regressions. First, we regressed teacher-perceived on observed aggressive student behavior. In the second step, we computed a multiple regression model to examine how chronic worry and resignation predict teachers' perceptions of aggression.

To answer our third research question, we z-standardized the two variables of teacher-perceived and observed aggressive student behavior. Then, we calculated the difference between the two z-standardized variables and implemented it as a new variable. This new variable was then regressed with chronic worry and resignation to determine whether these factors lead to underestimation or overestimation of perceived student aggression. Finally, we used multiple regressions to examine how perceived and observed aggressive student behavior predicted vital exhaustion and HCC.

## Results

### Observed and teacher-perceived student aggression

Means, standard deviations, and bivariate correlations between observed and perceived aggressive student behavior, chronic worry, resignation, vital exhaustion, HCC, and control variables are presented in Table 1.

**Table 1:** Descriptive statistics and Pearson intercorrelations of key variables

Variable	1	2	3	4	5	6	7	8	9
1. Observed aggression in the presence of the teacher	–								
2. Teacher-perceived aggression	.33*	–							
3. Chronic worry	.01	.55**	–						
4. Resignation	.04	.44**	.49**	–					
5. Vital exhaustion	.00	.55**	.74**	.58**	–				
6. HCC^a^	.20	.11	.09	− .12	.09	–			
7. Age	− .27	− .17	− .03	− .34*	− .14	.05	–		
8. Years of work experience	− .35*	− .20	.02	− .35*	− .01	.03	.83**	–	
9. Total lessons	.09	.31*	.07	.11	.18	.26	− .32*	− .20	–
10. Sex^b^	− .14	− .09	− .13	− .27	− .18	.18	.35*	.25	− .06
*M*^c^	14.24	1.82	1.15	15.19	35.95	7.77	39.66	13.35	23.07
*SD*^c^	27.34	0.65	0.95	4.50	9.99	7.71	11.99	11.07	3.97

*N* = 42 (28 females)

^a^*n* = 38*.* ^b^Sex: 0 = female 1 = male. ^c^*M* and *SD* are not log-transformed

^*^p < .05, two-tailed. **p < .01, two-tailed

Observed and teacher-perceived aggressive student behavior were significantly associated. Teacher-perceived aggression was positively correlated with chronic worry, resignation, and vital exhaustion. Observed aggression was not significantly associated with any of these variables. We observed less student aggression in the classes of teachers with more years of work experience. Experienced teachers reported less resignation than novice teachers. Teachers who teach a high amount of lessons per week reported more perceived student aggression. A simple linear regression was calculated to test whether observed aggression predicted teacher-perceived aggression. The standardized coefficient was β = 0.33, *p* = .036, explaining a small amount of variance [*F*(1, 40) = 4.73, *p* = .036, *R*^2^_adj_ = 0.08; Table 2].

**Table 2:** Regression results using teacher-perceived aggression as the criterion

Predictor	β	*SE*	95% CI		*p*	Fit
			*LL*	*UL*		
Model 1						
Observed student aggression	.325	.054	.008	.228	.036	
						R^2^_adj._ = .083
Model 2						
Chronic worries	.436	.118	.121	.599	.004	
Resignation tendency	.201	.011	− .007	.038	.169	
Total lessons	.259	.011	.000	.045	.045	
						R^2^_adj._ = .365

*N* = 42. β = standardized regression weights

*SE* standard error; *CI* confidence interval; *LL* lower limit; *UL* upper limit

### Avoidant coping styles and teacher-perceived aggression

We conducted multiple regression analyses to examine the effect of chronic worry and resignation on teacher-perceived aggression. The regression was controlled for total lessons. The regression model was significant [*F*(3, 38) = 8.85, *p* < .001, *R*^2^_adj_ = 0.37], indicating that the three predictors explained 37% of the variance in teacher-perceived aggression. While chronic worry (β = 0.44, *p* = .004) contributed significantly to the model, resignation did not (β = 0.20, *p* = .169). Table 2 shows the linear regression of observed on perceived aggression (model 1) and the multiple regression with chronic worry and resignation (model 2).

In the next step, we calculated the difference between teacher-perceived and observed student aggression by z-standardizing both variables and then subtracting observed from perceived student aggression, resulting in the new variable Δaggression. Then, Δaggression was regressed on chronic worry [*F*(1, 40) = 12.16, *R*^2^_adj_ = 0.21, *p* < .001, β = 0.48] and resignation [*F*(1, 40) = 5.55, *R*^2^_adj_ = 0.10, *p* = .023, β = 0.35]. Results show that chronic worry and resignation were positively associated with an overestimation of perceived student aggression. To examine whether there was an association between over- or underestimation of perceived aggression and vital exhaustion, we regressed vital exhaustion on Δaggression and obtained a standardized coefficient of β = 0.47, *p* = .002 [*F*(1, 40) = 11.38, *R*^2^_adj_ = 0.20]. This finding indicates that overestimating student aggression was positively associated with vital exhaustion.

### Teacher-perceived student aggression, vital exhaustion, and hair cortisol

Two multiple regressions were conducted to examine the association of perceived and observed student aggression with vital exhaustion and HCC. First, vital exhaustion was regressed on perceived and observed aggression [*F*(2, 39) = 9.78, *R*^2^_adj_ = 0.30, *p* < .001]. Perceived student aggression significantly affected vital exhaustion (β = 0.61, *p* < .001). Observed aggression was not associated with vital exhaustion (β = − 0.20, *p* = .158). The same predictors did not explain a significant amount of the variance in HCC [*F*(2, 35) = 0.75, *R*^2^_adj_ = 0.00, *p* = .481]. Neither perceived (β = 0.05, *p* = .803) nor observed student aggression (β = 0.18, *p* = .312) had a significant effect on HCC. The effects of perceived and observed student aggression on vital exhaustion (model 3) and HCC (model 4) are presented in Table 3.

**Table 3:** Regression results using vital exhaustion (Model 3) and hair cortisol (Model 4) as the criterion

Predictor	β	*SE*	95% CI		*p*	Fit
			*LL*	*UL*		
*Model 3 *(*vital exhaustion*)						
Observed student aggression	− .199	.037	− .128	.022	.158	
Perceived student aggression	.611	.102	.245	.657	< .001	
						R^2^_adj._ = .300
*Model 4 *(*hair cortisol*)						
Observed student aggression	.182	.141	− .141	.430	.312	
Perceived student aggression	.045	.392	− .698	.895	.803	
						R^2^_adj._ = .00

Model 3: *N* = 42, Model 4: *n* = 38. β = standardized regression weights

*SE* standard error; *CI* confidence interval; *LL* lower limit; *UL* upper limit

## Discussion

The present ambulatory assessment study focuses on observed student aggression in presence of the teacher and teacher-perceived aggressive student behavior. We aimed to explore the associations between two avoidant coping styles (i.e., chronic worry and resignation) and teachers' perceptions of student aggression. Finally, we examined how observed and teacher-perceived student aggression is associated with teachers' vital exhaustion and HCC.

### Hypothesis 1

That observed and teacher-perceived student aggression are only moderately associated, was supported. This finding is in line with a previous study (Scherzinger & Wettstein, 2019) using the same observation system and aligns with the synthesis of 16 studies on observed and teacher-perceived student aggression (Skiba, 1989) as well as a meta-analysis of Achenbach et al. (1987). Observers have an observational advantage over teachers, who must act under pressure to make numerous decisions while concurrently staying on top of complex classroom events. Consequently, a potential perceptional asymmetry between teachers and trained external observers should be considered when investigating and measuring classroom processes. Moreover, we found that older teachers were exposed to less (observed) student aggression and reported lower levels of resignation than their younger colleagues. This result is in line with a study by Levy and Khoury-Kassabri (2022), which shows that experienced teachers use more adaptive coping styles than less experienced teachers and feel more confident in coping with student aggression.

### Hypothesis 2

Assuming that the avoidant coping styles of chronic worry and resignation better predict teacher-perceived aggression than observed aggression, was also supported. In the regression models, observed aggression explained 10.5% and avoidant coping styles 36.5% of the explained variance when controlling for total lessons. Even considering only single predictors, coping styles are more associated with teacher perceptions than observed student aggression. Furthermore, we demonstrated that these avoidant coping styles were associated with an increased level of student aggression as perceived by the teachers. This finding points in a similar direction as studies showing that teachers' unfavorable personality traits, like neuroticism and poor teacher self-efficacy beliefs (Evers et al., 2004; Kokkinos et al., 2005; Tsouloupas et al., 2010), low core-self-evaluation (including global self-esteem, generalized self-efficacy, emotional stability, and internal locus of control; Schneider et al., 2022) and dysfunctional coping styles (Chan, 1998; van Dick & Wagner, 2001), are associated with higher burnout levels.

### Hypothesis 3

Presuming that teacher-perceived but not observed student aggression is associated with vital exhaustion, was also supported. Our study demonstrates that overestimating student aggression is positively associated with vital exhaustion. Student aggression is generally considered a primary source of teacher stress. However, our study suggests that for teachers' vital exhaustion, it is not primarily a question of how much observable student aggression occurs in the class but how teachers perceive student behavior.

### Hypothesis 4

Assuming that teacher-perceived student aggression is significantly associated with HCC, was not supported. Neither observed nor teacher-perceived student aggression showed significant associations with HCC. Moreover, vital exhaustion and HCC are not significantly associated. Compared to other adult samples, our participants show lower HCC values (Qi et al., 2014; Sauvé et al., 2007), indicating that our teachers are mainly healthy. The lacking correspondence between self-reported stress levels and HCC was also found in other studies and may be due to methodological differences or confounders (Kudielka et al., 2006). Empirically, Noser et al. (2018) found no associations between vital exhaustion and HCC. A review on self-reported stress and HCC revealed significant results in less than half of the studies (Gidlow et al., 2016), whereas a meta-analysis conducted by Stalder et al. (2017) showed no association. There are various possible explanations for this lack of association. First, vital exhaustion in our non-clinical sample of healthy teachers may not have reached a certain threshold to result in a significant association with HCC (van der Meij et al., 2018). Second, following Rohleder (2018), there may be individuals with high HCC and others with low HCC within a larger sample, depending on how long chronic stress has been experienced. Consequently, no significant relationship to self-reports can be found. Finally, the missing association between self-reported stress levels and HCC may be due to difficulties in perceiving one's own (physiological) state adequately (Campbell & Ehlert, 2012; Gidlow et al., 2016; Mauss & Robinson, 2009; Pennebaker, 1982; Stalder et al., 2017; Wettstein, 2012). The latter could lead to teachers suffering from unfavorable physiological stress consequences that endanger their health in the long run without realizing it. It is, therefore, essential to take physiological variables into account that may help reveal potentially unnoticed risk factors at an early stage.

It can be assumed that avoidant coping styles and the associated overestimation of aggressive student behavior have unfavorable consequences. Avoidant coping styles may affect teachers' psychological stress, classroom management behavior, as well as students' motivation and learning (Klusmann et al., 2016). When teachers use avoidant coping styles and overestimate student aggression, they perceive the school environment as generally hostile and can exacerbate student misbehavior, creating a significant additional stressor (Lewis et al., 2005). Thus, due to their dysfunctional coping styles, they may become co-constructors of an increasingly hostile environment characterized by highly aggressive students (in their eyes), which in turn increases teachers' risk for vital exhaustion and inappropriate classroom management behavior. Teachers may be caught in a vicious cycle of distorted perceptions, dysfunctional coping styles in response to alleged student misbehavior, and subsequent actual student misbehavior. This line of thought is consistent with a study by Kokkinos et al. (2005), who demonstrated that teachers' level of emotional exhaustion increased the appraised severity of students' externalizing behaviors. Further, previous studies have shown that teachers' negative feelings about student aggression may result in counterproductive classroom management behavior, such as direct confrontation and defensive reactions (Chang & Davis, 2009).

### Implications

Student aggression is considered a primary source of teacher stress in the classroom, endangering teachers' health. Consequently, teacher education and professional development have traditionally emphasized improving teachers' management of student misbehavior. However, this approach may fall short. Our study shows that it is not the student aggression per se that associates with teachers' vital exhaustion but how teachers perceive it. Therefore, teacher education must also emphasize teachers' coping styles when facing student misbehavior. It is critical to sensitize teachers to their own avoidant coping styles that may distort the perception of aggressive student behavior and foster the use of proactive problem-focused coping styles. Teachers should be encouraged to confront and appraise stressors in teaching as a challenge and learn to manage stress actively (Chan, 1998). Some universities already train teachers in adaptive coping styles in basic training and continuing education. If we succeed in bundling these efforts systematically and collaboratively, it may be possible to counteract teachers' unfavorable coping styles more efficiently.

### Limitations and strengths

The present study has some limitations. First, the findings are based on a small convenience sample of apparently healthy and medication-free teachers and cannot be generalized to the entire population. Second, we examined the effects of chronic worry on perceived student aggression. However, perceived student aggression could also influence chronic worry, making a bidirectional causality possible. Third, we only used cross-sectional data, further restricting conclusions on causal relations. Fourth, in assessing aggressive student behavior from the teacher and observers' perspective, the period to which the observations referred necessarily differed. The coding of the external observers was limited to videotaped lessons, while the teacher's assessments referred to aggressive behavior in general in this class.

Despite these limitations, the present study highlights the value of a multi-method approach, which combines self-reports, physiological data, and objective behavioral observation in an ambulatory setting, compared to relying solely on self-reports.

Further research could examine a broader range of coping styles, teacher personality traits, and classroom management behaviors in a longitudinal design and thus substantially improve our understanding of the central role of coping styles in dealing with student misbehavior and teacher stress. Furthermore, future studies could systematically analyze teachers' acute physiological stress reactions during specific episodes of student aggression.

## Conclusion

There is a broad consensus that aggressive student behavior is associated with teacher stress. While most previous studies assessing aggressive student behavior were limited to self-reports, this study goes one step further and includes systematic behavioral observation as well as physiological measures. Our findings suggest that teachers do not perceive “objective” student aggression directly but do so through the lens of their potentially dysfunctional coping styles. It seems that teachers' dysfunctional coping styles distort teachers' perceptions, resulting in an overestimation of aggressive student behavior which, in turn, is associated with increased levels of vital exhaustion. Teachers' dysfunctional coping styles not only stigmatize students but also directly endangers teacher health. Therefore, it is crucial to promote teachers' use of proactive problem-focused coping styles.

## Data Availability

The data that support the findings of this study are available from the corresponding author upon reasonable request.
